# Sesamin Protects against and Ameliorates Rat Intestinal Ischemia/Reperfusion Injury with Involvement of Activating Nrf2/HO-1/NQO1 Signaling Pathway

**DOI:** 10.1155/2021/5147069

**Published:** 2021-09-29

**Authors:** Yilin Wang, Jin Wen, Marwan Almoiliqy, Yaojia Wang, Zhihao Liu, Xiaobo Yang, Xiaolong Lu, Qiang Meng, Jinyong Peng, Yuan Lin, Pengyuan Sun

**Affiliations:** ^1^College of Pharmacy, Dalian Medical University, Dalian, 116044 Liaoning, China; ^2^Affiliated Zhongshan Hospital of Dalian University, Dalian, 116001 Liaoning, China; ^3^Key Lab of Aromatic Plant Resources Exploitation and Utilization in Sichuan Higher Education, Yibin University, Yibin, 644000 Sichuan, China; ^4^Key Laboratory for Basic and Applied Research on Pharmacodynamic Substances of Traditional Chinese Medicine of Liaoning Province, Dalian Medical University, Dalian, 116044 Liaoning, China

## Abstract

Intestinal ischemia-reperfusion (I/R) may induce cell/tissue injuries, leading to multiple organ failure. Based on our preexperiments, we proposed that sesamin could protect against and ameliorate intestinal I/R injuries and related disorders with involvement of activating Nrf2 signaling pathway. This proposal was evaluated using SD intestinal I/R injury rats *in vivo* and hypoxia/reoxygenation- (H/R-) injured rat small intestinal crypt epithelial cell line (IEC-6 cells) *in vitro*. Sesamin significantly alleviated I/R-induced intestinal histopathological injuries and significantly reduced serum biochemical indicators ALT and AST, alleviating I/R-induced intestinal injury in rats. Sesamin also significantly reversed I/R-increased TNF-*α*, IL-6, IL-1*β*, and MPO activity in serum and MDA in tissues and I/R-decreased GSH in tissues and SOD in both tissues and IEC-6 cells, indicating its anti-inflammatory and antioxidative stress effects. Further, sesamin significantly decreased TUNEL-positive cells, downregulated the increased Bax and caspase-3 protein expression, upregulated the decreased protein expression of Bcl-2 in I/R-injured intestinal tissues, and significantly reversed H/R-reduced IEC-6 cell viability as well as reduced the number of apoptotic cells among H/R-injured IEC-6 cell, showing antiapoptotic effects. Activation of Nrf2 is known to ameliorate tissue/cell injuries. Consistent with sesamin-induced ameliorations of both intestinal I/R injuries and H/R injuries, transfection of Nrf2 cDNA significantly upregulated the expression of Nrf2, HO-1, and NQO1, respectively. On the contrary, either Nrf2 inhibitor (ML385) or Nrf2 siRNA transfection significantly decreased the expression of these proteins. Our results suggest that activation of the Nrf2/HO-1/NQO1 signaling pathway is involved in sesamin-induced anti-inflammatory, antioxidative, and antiapoptotic effects in protection against and amelioration of intestinal I/R injuries.

## 1. Introduction

Intestinal ischemia-reperfusion (I/R) can be caused by acute mesenteric ischemia, severe infection, traumatic shock, and surgical procedures, leading to multiple organ dysfunction and systemic inflammatory response syndrome [1, 2]. And until now, intestinal I/R injuries still represent a great challenge in clinical practice, with high morbidity and mortality [3].

**Figure 1:**
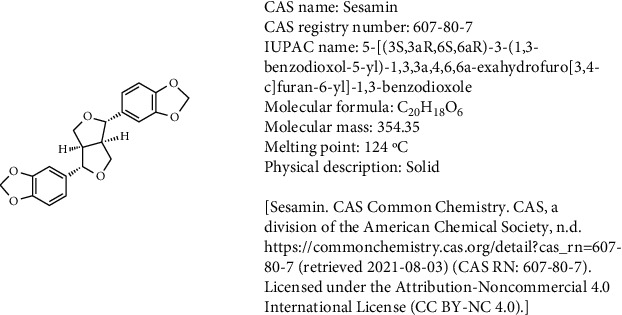
Chemical structure of sesamin. Sesamin is a lignan that consists of tetrahydro-1H,3H-furo[3,4-c]furan substituted by 1,3-benzodioxole groups at positions 1 and 4 (the 1S,3aR,4S,6aR stereoisomer). Isolated from Cinnamomum camphora, it exhibits cytotoxic activity. It has a role as an antineoplastic agent, a neuroprotective agent, and a plant metabolite. It is a lignan, a member of benzodioxoles, and a furofuran [PubChem [Internet]. Bethesda (MD): National Library of Medicine (US), National Center for Biotechnology Information; 2004-. PubChem Compound Summary for CID 72307, Sesamin; [cited 2021 Aug. 3]. Available from: https://pubchem.ncbi.nlm.nih.gov/compound/Sesamin].

Sesamin (Figure 1), a natural lignin compound, is isolated from sesame seeds, and it possesses health beneficial effects, including anti-inflammatory, anticancer, antihypertensive, antithrombotic, antidiabetic, antiatherogenic, antiobesity, and lipolytic effects [4, 5], and ameliorates intestinal, renal, cardiac, brain, and hepatic injury [6–11]. However, the key modulatory properties and characteristics of sesamin and related mechanisms in ameliorating intestinal I/R injury remain to be explored.

Based on our preliminary experiments, we proposed that sesamin pretreatment could protect against and ameliorate I/R-induced injuries by reducing the oxidative stress, inflammation, and apoptosis with involvement of the activation of nuclear factor erythroid 2-related factor 2 (Nrf2) signaling pathway. Intestinal I/R injury rat model *in vivo* and rat small intestinal crypt epithelial cells (IEC-6) treated with hypoxia-reoxygenation (H/R) model *in vitro* were used to jointly verify our proposal.

## 2. Materials and Methods

### 2.1. Chemicals and Materials (Table 1: Supplementary Material S1)

Sesamin (purity: ≥98%) was bought from Dalian Meilun Biotech Co., Ltd. (Dalian, China). The test kits for aspartate aminotransferase (AST), alanine aminotransferase (ALT), myeloperoxidase (MPO), malondialdehyde (MDA), superoxide dismutase (SOD), glutathione (GSH), and ML385 were provided by Nanjing Jiancheng Institute of Biotechnology (Nanjing, China). The detection boxes for double-antibody sandwich ELISAs of interleukin-1*β* (IL-1*β*), interleukin-6 (IL-6), and tumor necrosis factor *α* (TNF-*α*) were supplied by ShangHai Lengton Bioscience Co. Ltd. (Shanghai, China). Dulbecco's minimal essential medium (DMEM) and fetal bovine serum (FBS) were purchased from Hyclone Laboratory (CA, USA) and Gibco (CA, USA), respectively. The terminal deoxynucleotidyl transferase-mediated dUTP-biotin nick-end labeling (TUNEL) kit was supplied by Servicebio Company (Wuhan, China). The cell counting kit-8 (CCK-8) was provided by Bio-Tool (Beijing, China). siRNA of Nrf2 and Lipofectamine 2000 were bought from GenePharma (Shanghai, China). The rat small intestinal crypt epithelial cell line (IEC-6 cells) was purchased from the company of American Tissue Culture Collection (ATCC, USA). Unless otherwise indicated, the chemicals were bought from Sigma-Aldrich (St. Louis, MO, USA). All the chemicals used in the study were analytical grade reagents.

### 2.2. Animals

Sprague-Dawley (SD) rats (Male) weighing 200-220 g were supplied by the Experimental Animal Center of Dalian Medical University (Certificate of Conformity: No. SCXK (Liao) 2018-0003). All the experimental protocols were seriously carried out in accordance with the guidelines issued by the National Institutes of Health Guide for Care and Use of Laboratory Animals (No. 85-23, revised in 1985) and the Animal Care and Ethics Committee of Dalian Medical University (No. L20140402). Two weeks before the experiment, the rats were adapted to the laboratory room temperature (23°C), 12 h of light and 12 h of darkness, 50% indoor humidity, and free food and water. The design of animal experiment program can minimize the discomfort and pain to the rats. Before experiment *in vivo*, rats were raised separately (one rat per cage) and fasted for 12 h.

### 2.3. Establishment of Rat Intestinal I/R Injury Model

The rat model of intestinal I/R was established according to the protocol described previously [12]. After one week of feeding, rats were fasted for 12 h and were allowed to drink water freely before establishing intestinal I/R injury. Rats were anesthetized via intraperitoneal injection of pentobarbital (50 mg/kg body weight), and then, abdominal incision was made. After the superior mesenteric artery (SMA) was isolated, the traumatic microvascular clamp was used to block it for 1 h to induce ischemia, and then, the clamp was removed for 2 h of reperfusion. Rats were euthanized, and 8 cm long jejunum sections were collected after reperfusion was finished. Part of jejunum samples was fixed with formalin (4% paraformaldehyde solution) for hematoxylin and eosin (H&E), TUNEL, and histological analysis.

The doses of sesamin (5, 15 mg/kg) were determined based on the information from the reference literatures [13, 14] and our preexperiments. Sesamin was suspended in sodium carboxymethyl cellulose (0.5% CMC-Na) and used in the study. The sham-operated and intestinal I/R-injured rats were administrated with the same volume of CMC-Na.

**Figure 2:**
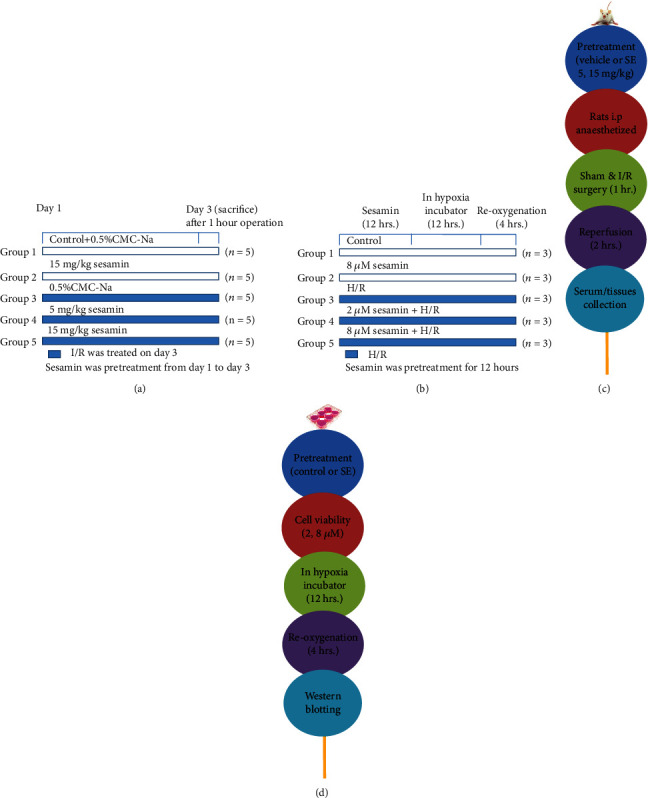
Schematic of experimental design and methods used in the study: (a) *in vivo* experimental design and (c) methods using SD intestinal I/R injury rats; (b) *in vitro* experimental design and (d) methods using H/R-injured IEC-6 cells.

Rats were randomly assigned into five groups (*n* = 5 in each group, Figures 2(a) and 2(c)): (1) sham operation group: rats were given vehicle by gavage for 3 consecutive days and followed by 1 h exposure of the mesenteric artery with no ligation; (2) sesamin (H)+sham operation group: rats were treated with sesamin at a dose of 15 mg/kg before sham operation; (3) I/R group: rats were given vehicle by gavage for 3 consecutive days and then for 1 h of intestinal ischemia and 2 h of reperfusion; (4) sesamin (L)+I/R group: rats were treated with sesamin at a dose of 5 mg/kg prior to I/R induction; and (5) sesamin (H)+I/R group: rats were treated with sesamin at a dose of 15 mg/kg before I/R induction.

### 2.4. Histological Staining

Formalin-fixed rat intestine segments were embedded in paraffin and then sectioned (4 *μ*m slice) using sectioning apparatus (Leica, RM2245, Germany). H&E staining was performed on randomly selected intestinal tissue samples according to the manufacturer's instruction, and Chiu's score evaluation was used to determine the degree of intestinal I/R injury [15].

### 2.5. Biochemical Analysis

After 20 min of natural coagulation at room temperature, blood samples were centrifuged (4°C, 20 min, 3000 × g/min) and the serum was collected. Intestinal tissues were homogenized in ice-cold normal saline, and the supernatant was collected after centrifugation (4°C, 15 min, 3500 × g/min). ELISA kits were used to measure proinflammation cytokines and mediators (IL-6, IL-1*β*, and TNF-*α*). Commercial kits were used to determine AST and ALT in serum and MDA, SOD, GSH, and MPO in the supernatant.

### 2.6. Cell Culture and Hypoxia/Reoxygenation (H/R) Incubation

Under the external environment (37°C, 5% CO_2_) recommended by the supplier, IEC-6 cells were incubated in DMEM supplemented with 10% FBS, bovine insulin (0.1 U/mL), and double antibodies (50 U/mL penicillin and 50 U/mL streptomycin) [16]. In order to induce hypoxia, IEC-6 cells were transferred into a humidified hypoxia chamber (Thermo, Waltham, MA) containing 1% O_2_ and 5% CO_2_, balanced with 94% N_2_ for 12 h, and then cultured for 4 h under normoxic conditions (Figures 2(b) and 2(d)) [17].

### 2.7. Cell Viability Assay

CCK-8 assay was performed to evaluate IEC-6 cell viability. Before H/R induction, IEC-6 cells were seeded in 96-well plates (100 *μ*L/well, 1 × 10^4^ cells) and allowed to adhere. Then, IEC-6 cells were administrated with gradient dilution of sesamin (1, 2, 4, 8, 16, and 32 *μ*M) and treated with 5% CO_2_ for 12 h (37°C). After H/R induction, 10% CCK-8 was dissolved in DMEM and then added to each well (100 *μ*L). CCK-8 was incubated at 37°C for 1 h, and optical densities were read at 450 nm with an enzymograph (Enspire 2300).

### 2.8. Cell Transfection

IEC-6 cells (1 × 10^5^) were transfected by small interfering RNA (siRNA) or cDNA [18] and seeded in 6-well plates for 24 h. Specific siRNA (GenePharma, Shanghai, China) or cDNA (GenePharma, Shanghai, China) targeting Nrf2 were transfected into cells with Lipofectamine 2000. After 6 h of transfection, the cells were incubated under H/R condition or incubated under H/R condition pretreated with sesamin (8 *μ*M) for another continuous 48 h and then analyzed using Western blotting.

### 2.9. Measurement of Morphological Alteration

IEC-6 cells were seeded into 6-well plates for 24 h (37°C) with a single-cell suspension containing 10% FBS. The cells were incubated with sesamin (8 *μ*M) for 12 h, placed in hypoxia incubator for 12 h, and then developed for 4 h to achieve reoxygenation under normoxia condition. ICE-6 cells were rinsed twice in PBS after incubation and randomly distributed to the protection group and the sham group. The image of the growth of IEC-6 cells in each group was caught using an inverted microscope (Nikon, Japan), and morphological changes were evaluated after the cell-drug interaction. Fluorescence staining of DAPI was performed in a similar way, and IEC-6 cells were fixed in 10% paraformaldehyde (10 min). Cells were stained with DAPI (1 *μ*g/mL) in the dark at room temperature for 10 min and then rinsed in PBS for three times. According to the instruction of manufacturer, ROS evaluation was detected by ROS staining kits (Beyotime, Jiangsu, China). IEC-6 cells were plated overnight, and sesamin was added before H/R for 12 h. Then, the cells were rinsed in PBS and treated with an ROS solution for 20 min (37°C). Finally, the images were caught by inverted fluorescence microscopy (BX63, IX81, Olympus, Japan) and the pictures were recorded.

### 2.10. Immunostaining of TUNEL

Dewaxed tissue samples were incubated with 0.1% Triton X-100 (10 min). Then, the incubated samples were rinsed in PBS. The rinsed samples were stained with TUNEL-based Apoptosis Detection Assay (Nanjing Jiancheng Corp, China) kits according to the instruction of manufacturer. Apoptotic cells were determined by counting the average number of TUNEL-positive cells (green spots) using fluorescence microscopy (BX63, IX81, Olympus, Japan).

### 2.11. Western Blotting Analysis

According to the instruction of manufacturer, total protein was extracted from intestinal tissues and IEC-6 cells, respectively. The supernatant was collected and quantified using a Coomassie Brilliant Blue G-250 kit (Solarbio, Beijing, China). 10% SDS-PAGE gel was used to separate the protein samples (25 *μ*g), and the separated samples were transferred to PVDF membrane. 5% skimmed milk or 5% bovine serum albumin was diluted in Tris-buffered saline containing Tween (TBS-T) to seal the membranes (3 h, 37°C). And the sealed membranes were hatched overnight (4°C) with following primary antibodies: Bcl-2-associated X (Bax), B-cell lymphoma-2 (Bcl-2), cysteinyl aspartate specific proteinase 3 (caspase-3), SOD-2, Nrf2, heme oxygenase 1 (HO-1), NAD(P)H quinone dehydrogenase 1 (NQO1), and *β*-actin (Proteintech, Wuhan, China). After the first antibody was incubated, the imprints were rinsed in TBS-T for three times and then were hatched (room temperature, 2 h) with a horseradish-conjugated goat anti-rabbit antibody (Proteintech, Wuhan, China). ECL detection system was used to detect protein abundance. Image Lab software (Bio-Rad, CA, USA) was used to measure protein quantitation in optical density units and standardized to the corresponding *β*-actin sample expression.

### 2.12. Statistical Analysis

Data were analyzed using GraphPad Prism 5.0 (GraphPad Prism Software, La Jolla, California). All values are expressed as mean ± SEM. One-way analysis of variance (ANOVA) followed by the Student-Newman-Keuls (SNK) test was used to the normal distribution data. All experimental results were obtained from at least 3 independent experiments. Investigating 3 animals per group (as stated for many parameters) is really a great limitation for animal experimental research. Statistically significant differences were shown as *P* < 0.05.

## 3. Results

### 3.1. Sesamin-Induced Protection against Intestinal I/R Morphological Injuries

**Figure 3:**
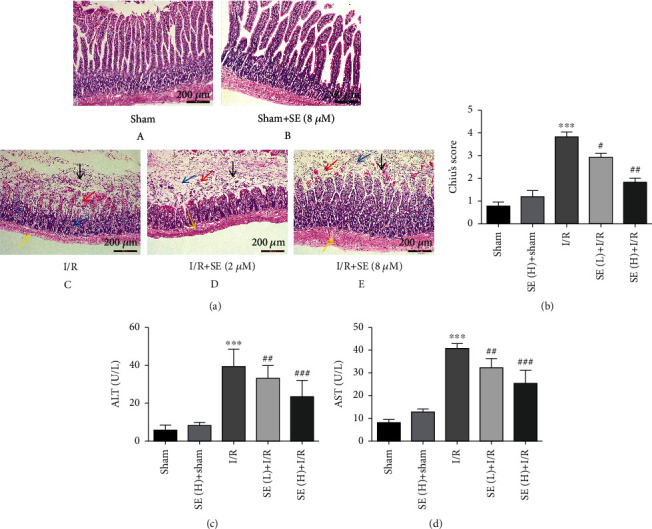
Effects of sesamin on I/R-induced histological alteration in rat intestine. (a) Representative images of pathological changes of intestinal mucosal tissues (H&E staining, original magnification ×200). (A) Sham operation group; (B) SE(H)+sham operation group; (C) I/R group: a large number of intestinal villi epithelial cells fall off in the mucosal layer (black arrow), more intestinal villi become blunt, the mucosal layer is slightly edema, connective tissue is loosely arranged, the distance between intestinal glands is widened (blue arrow), and a small number of lymphocytes are scattered (red arrow); a small amount of lymphocyte infiltration can be seen in the submucosa and serosal layer (yellow arrow); (D) SE(L)+I/R group and (E) SE(H)+I/R group: after sesamin pretreatment, the intestinal mucosal damage has been significantly improved with the increase of the dose and the inflammatory cells and bleeding are reduced. (b) Chiu's score for the damage of the intestinal mucosa (*n* = 3). (c) Serum ALT (*n* = 5) and (d) serum AST (*n* = 5). All data were expressed as mean ± SEM. ^∗∗∗^*P* < 0.001*vs.* the sham-operation group; ^#^*P* < 0.05, ^##^*P* < 0.01, and ^###^*P* < 0.01*vs.* the I/R group.

The intestinal mucosal barrier and goblet cells in sham-operated (Figure 3(a), A) and sesamin+sham-operated rats (Figure 3(a), B) were intact and visible, and the villi were arranged in order. Compared with the sham-operated rats, the mucosal epithelial cell layers were necrotic and shedding, villi were broken, and interstitial fracture, inflammatory cell infiltration, scattered ulcers, and hemorrhage were observed in I/R-injured rats (Figures 3(a), C). As shown in Figure 3(a), D and E, sesamin (5, 15 mg/kg) significantly protected against and reduced I/R-induced intestinal injury. Chiu's score showed that the injury degree of intestinal mucosa in I/R-injured rats was more severe than that in sham-operated rats, and the increased Chiu's score in I/R-injured rats was significantly reversed by sesamin (Figure 3(b)). Sesamin significantly decreased serum ALT and AST level, which are biochemical indicators of remote organ injuries, without altering these parameters in sham-operated rats (Figures 3(c) and 3(d)), suggesting sesamin-induced protection on remote organs.

### 3.2. Protection of Sesamin against Intestinal I/R-Induced Inflammation and Oxidative Stress

**Figure 4:**
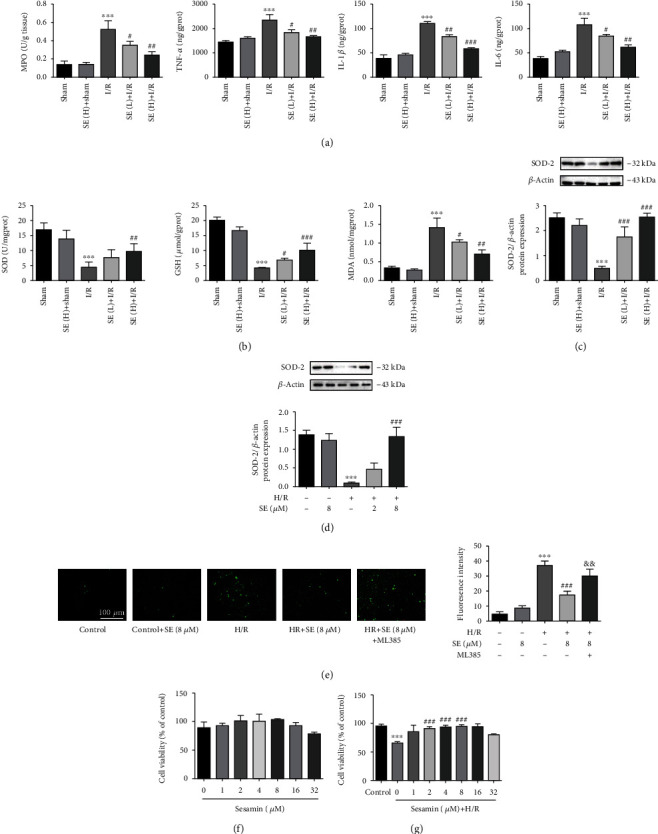
Protection of sesamin against intestinal I/R-induced inflammation and oxidative stress: (a) MPO in intestinal tissues and TNF-*α*, IL-1*β*, and IL-6 in serum (ELISA kits) (*n* = 5); (b) SOD, GSH, and MDA in intestinal tissue homogenate supernatant (commercial kits) (*n* = 5); (c) SOD protein expression in intestinal tissues in each group (*n* = 3); (d) SOD protein expression in normal (*n* = 3) and H/R-injured IEC-6 cells (*n* = 3); (e) the fluorescence images for oxidative changes of IEC-6 cells (*n* = 3); (f) different concentrations of sesamin pretreated on IEC-6 cells for 12 h under normal condition (*n* = 3); (g) different concentrations of sesamin pretreatment on IEC-6 cells in 12 h under H/R conditions (*n* = 3). Values are expressed as mean ± SEM. ^∗∗^*P* < 0.01 and ^∗∗∗^*P* < 0.001*vs.* the sham group; ^#^*P* < 0.05, ^##^*P* < 0.01, and ^###^*P* < 0.001*vs.* the I/R and H/R group.

Compared with sham-operated rats, the proinflammatory cytokines (MPO, TNF-*α*, IL-1*β*, and IL-6) were significantly elevated in the serum of I/R-injured rats. Sesamin (5, 15 mg/kg) significantly reversed the increased cytokines in a dose-dependent manner (Figure 4(a)), indicating that sesamin-reduced intestinal injuries were related to its anti-inflammatory effects. In sham-operated rats, these parameters were not significantly influenced by sesamin. Compared with sham-operated rats, downregulation of SOD and GSH (biomarkers of antioxidant capacity) and upregulation of MDA (a biomarker of oxidative damage) were found in tissue homogenate supernatant of I/R-injured rats. Sesamin significantly reversed these biomarkers in a dose-dependent manner (Figure 4(b)). Compared with the corresponding normal controls, a significant decrease of SOD protein expression was found in both I/R-injured intestinal tissues (Figure 4(c)) and H/R-injured IEC-6 cells (Figure 4(d)) using Western blotting analysis, and the decreased protein expression was significantly reversed by sesamin (Figures 4(c) and 4(d)). Compared with the corresponding control IEC-6 cells, a significant increase in ROS generation was found in H/R-injured IEC-6 cells using fluorescence assay, and this increase in ROS was significantly reversed by sesamin (Figure 4(e)), suggesting that sesamin-reduced intestinal I/R injury was related to the inhibition of oxidative stress. Nrf2 activation is related to suppression of oxidative stress. Nrf2 inhibitor ML385 abolished the reversal induced by sesamin on H/R-injured IEC-6 cells. These results suggested that the protection of sesamin against intestinal I/R injury is related to the suppression of oxidative stress with involvement of activation of the Nrf2 signaling pathway.

### 3.3. Protection of Sesamin on Cell Viability

As shown in Figure 4(f), the viability of normal IEC-6 cells was not significantly affected by sesamin in a concentration up to 16 *μ*M after 12 h incubation. Although cell viability was inhibited by sesamin at 32 *μ*M, no significant decreased cell viability was observed. Similar to our study, Chen et al. [19] and Xu et al. [20] reported that no significant decreased cell viability was observed in human oral cancer cell lines and neuroblastoma cell lines at 40 *μ*M and 50 *μ*M, respectively. Thus, 2-8 *μ*M sesamin was selected as the appropriate dose range for subsequent *in vitro* study. H/R injury significantly reduced the viability of normal ICE-6 cells, and sesamin pretreatment (2, 4, and 8 *μ*M for 12 h) significantly reversed this decrease compared with H/R-injured cells (Figure 4(g)), suggesting that sesamin-exerted selective reversal of the decreased IEC-6 cell viability was involved in the protection against H/R-induced injuries. It is worth noting that sesamin (32 *μ*M) did not reverse the decreased cell viability, which indicates that there is a phenomenon similar to normal cells; that is, high concentration of sesamin inhibited the cell viability.

### 3.4. Protection of Sesamin against I/R- and H/R-Induced Apoptosis

**Figure 5:**
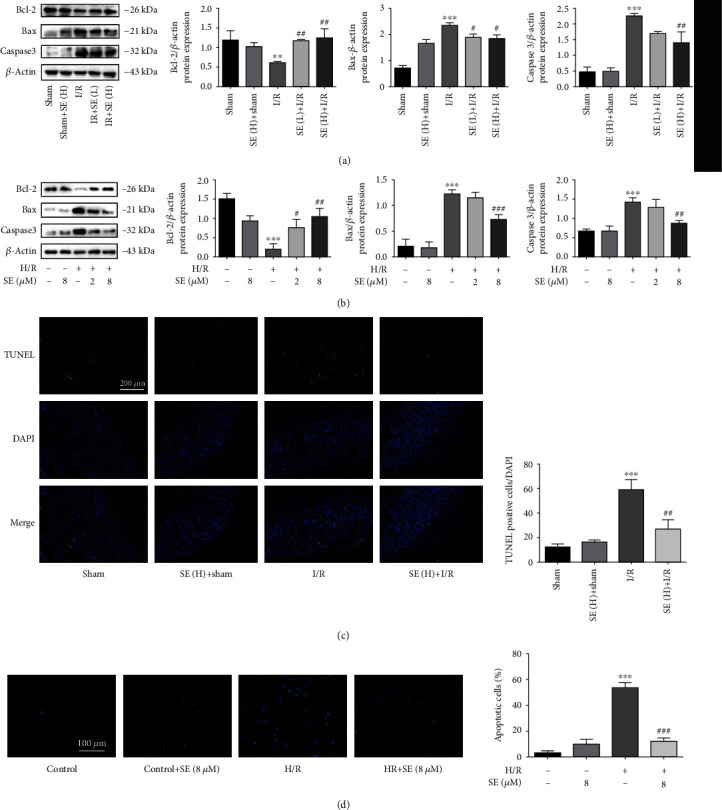
Western blotting analysis of sesamin against intestinal I/R- and H/R-induced apoptosis: (a) intestinal Bcl-2, Bax, and caspase-3 protein expression in the sham, sham+SE (H), I/R, I/R+SE (L), and I/R+SE (H) group (*n* = 3); (b) Bcl-2, Bax, and caspase-3 protein expression in the IEC-6 cell, IEC-6 cell+SE, H/R-injured IEC-6 cell, I/R-injured IEC-6 cell+SE (2 *μ*M), and I/R-injured IEC-6 cell+SE (8 *μ*M) group (*n* = 3); (c) TUNEL staining of intestinal tissues before and after I/R injury in the absence and presence of sesamin (*n* = 3); (d) the fluorescence images of apoptosis of IEC-6 cells before and after H/R injury and in the absence and presence of sesamin (*n* = 3). Values are expressed as mean ± SEM. ^∗^*P* < 0.05, ^∗∗^*P* < 0.01, and ^∗∗∗^*P* < 0.001*vs.* the sham group; ^#^*P* < 0.05, ^##^*P* < 0.01, and ^###^*P* < 0.001*vs.* the I/R and H/R group.

Apoptosis is a major cause of cell death in I/R injury [21, 22]. Bcl-2 family, including Bax, and caspase family are the core regulators of the intrinsic pathway of apoptosis [21–26]. As shown in Figures 5(a) and 5(b), Bcl-2 was significantly decreased and both Bax and caspase-3 were significantly increased in both I/R-injured intestinal tissues and H/R-injured IEC-6 cells, and these parameters were significantly reversed by using sesamin (5, 15 mg/kg).

I/R induces DNA damage. TUNEL assays can be used for histochemical localization of apoptosis with in situ labeling of fragmented DNA [27]. Compared with I/R-injured rats, sesamin (15 mg/kg) significantly reduced TUNEL signals in the intestinal tissues, indicating that sesamin decreased DNA damages in cells (Figure 5(c)). No significant effects on TUNEL signals were found in sesamin (H)+sham rats.

DAPI staining is used to detect apoptosis *in vitro* [22, 28]. The *in situ* observation in the IEC-6 battery configuration is shown in Figure 5(d). The chromatin of the IEC-6 cells was concentrated and appeared to be bright blue in the apoptotic positive cells, compared to the normal IEC-6 cell control. And sesamin significantly reduced the number of the bright blue cells, suggesting sesamin-induced antiapoptotic effects.

### 3.5. Sesamin-Induced Activation of the Nrf2 Signaling Pathway in Protecting against Intestinal I/R Injuries

The protective role of Nrf2 activation with involvement of its downstream pathways such as HO-1 and NQO1 is characterized by inhibiting inflammation, oxidative stress, and apoptosis [29–31], which are involved in ameliorating kidney [32], brain [33], and myocardial injuries [34].

#### 3.5.1. Effects of Sesamin on Nrf2/HO-1/NQO1 Protein Expression

**Figure 6:**
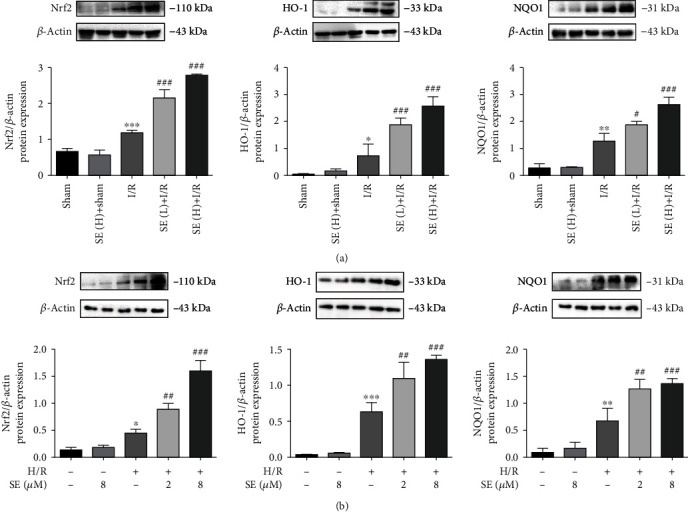
Western blotting analysis of protein expression: (a) Nrf2/HO-1/NQO1 protein expression in the sham, sham+SE (H), I/R, I/R+SE (L), and I/R+SE (H) group (*n* = 3); (b) Nrf2/HO-1/NQO1 protein expression in the IEC-6 cell, IEC-6 cell+SE, H/R-injured IEC-6 cell, I/R-injured IEC-6 cell+SE (2 *μ*M), and I/R-injured IEC-6 cell+SE (8 *μ*M) group (*n* = 3). Data are expressed as mean ± SEM. ^∗^*P* < 0.05, ^∗∗^*P* < 0.01, and ^∗∗∗^*P* < 0.001*vs.* the sham group; ^#^*P* < 0.05, ^##^*P* < 0.01, and ^###^*P* < 0.001*vs.* the H/R group. SE: sesamin.

Our results indicated that Nrf2, HO-1, and NQO1 protein expression was significantly increased in I/R-injured intestinal tissues (Figure 6(a)) and in H/R-injured IEC-6 cells (Figure 6(b)), compared with their respective normal controls. The protective role of Nrf2 in injury amelioration was also observed and verified in the presence of sesamin. Sesamin further increased the protein expression of Nrf2, HO-1, and NQO1, compared with their respective I/R-injured intestinal tissues (Figure 6(a)) and H/R-injured IEC-6 cells (Figure 6(b)).

#### 3.5.2. Effects of Nrf2 cDNA on the Expression in the Nrf2/HO-1/NQO1 Signaling Pathway

**Figure 7:**
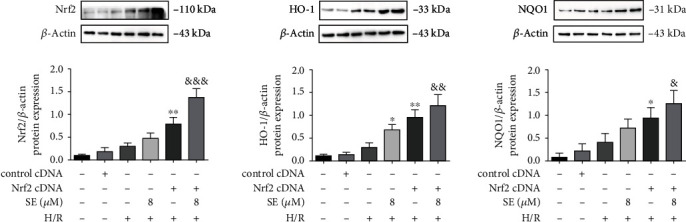
Effects of Nrf2 cDNA amplification on Nrf2, HO-1, and NQO1 protein expression in the absence and presence of sesamin (8 *μ*M) in normal and H/R-injured IEC-6 cell. Values are expressed as mean ± SEM (*n* = 3). ^∗^*P* < 0.05 and ^∗∗^*P* < 0.01 vs. the H/R group; ^&^*P* < 0.05, ^&&^*P* < 0.01, and ^&&&^*P* < 0.001 vs. H/R and sesamin.

Data from Nrf2 cDNA transfections (Figure 7) indicated that either sesamin or overexpression of Nrf2 increased Nrf2, HO-1, and NQO1 protein expression in H/R-injured IEC-6 cell, and the cDNA transfection+sesamin further increased Nrf2, HO-1, and NQO1 protein expression in H/R-injured IEC-6 cell.

#### 3.5.3. Nrf2 Inhibitor (ML385) and Nrf2 siRNA on Nrf2 Protein Expression in the Absence/Presence of Sesamin

**Figure 8:**
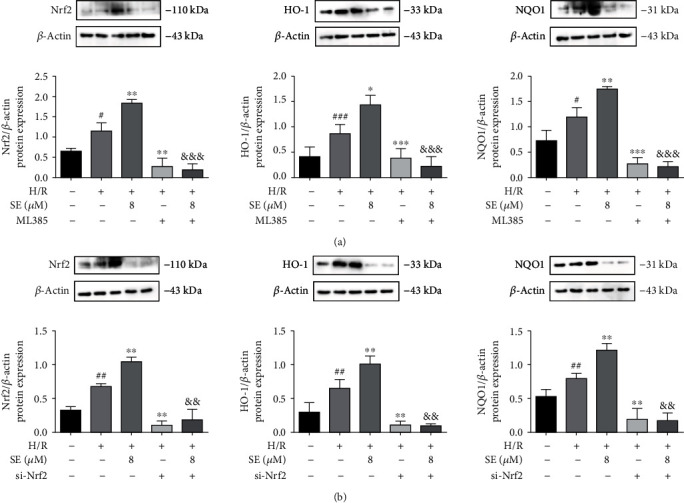
Western blotting analysis of the effects of Nrf2 inhibitor ML385 or Nrf2 siRNA on Nrf2, HO-1, and NQO1 protein expression in the absence or presence of sesamin in normal and H/R-injured IEC-6 cells. (a) ML385 (10 *μ*M) and (b) siRNA Nrf2 were used, respectively, to evaluate their relationships with sesamin (SE) (8 *μ*M) in modulating H/R-injured IEC-6 cells (*n* = 3). Values are expressed as mean ± SEM. ^##^*P* < 0.01 and ^###^*P* < 0.001*vs.* the sham group; ^∗^*P* < 0.05, ^∗∗^*P* < 0.01, and ^∗∗∗^*P* < 0.001*vs.* the H/R group; ^&&^*P* < 0.01 and ^&&&^*P* < 0.001*vs.* H/R and sesamin.

To verify whether the protective effects of sesamin on H/R injury were related to Nrf2 activation, ML385 (an Nrf2 inhibitor) and si-Nrf2 were used, respectively, in the study. As shown in Figure 8, Nrf2/HO-1/NQO1 protein expression in H/R-injured IEC-6 cells was significantly increased, and sesamin further enhanced the expression of these proteins. ML385 and si-Nrf2, respectively, downregulated Nrf2/HO-1/NQO1 protein expression in H/R-injured IEC-6 cells in both the presence and absence of sesamin in H/R-injured IEC-6 cells.

## 4. Discussion

Several mechanisms are involved in intestinal I/R injury, such as microvascular dysfunction [35], excessive production of reactive oxygen species [36–38], inflammation [39–41], and cell apoptosis [42–44]. Histological evidence of intestinal I/R injury includes decreased villus height, increased cellular infiltrate, and mucosal sloughing [45]. And the increase of serum aminotransferase levels, such as AST and ALT, caused by remote organ injuries is also one of the characteristics of intestinal I/R injury [28, 46, 47]. Proinflammatory cytokines, such as TNF-*α*, IL-6, and IL-1*β*, in serum of intestinal I/R animals are significantly increased [22, 28, 48], which is related to neutrophil activation and tissue damage caused by I/R [47, 49]. MPO content is found to reflect neutrophil accumulation and activity [16, 50]. Generation of ROS is involved in the progression of the intestinal I/R injury [47, 51], and the antioxidant system may compensate for these damages caused by ROS through enzymatic substances, such as SOD, and nonenzymatic substances, such as GSH [22, 28]. Intestinal I/R injury is also found to cause extensive cell apoptosis [16, 52]. Inhibition of cell death mechanism by blocking apoptotic markers (Bax and caspase 3/9) [22, 28, 53–55] or overexpressing antiapoptotic markers (Bcl2) [22, 28, 54–56] prevents the pathogenesis of intestinal I/R injury. Therefore, compounds with multiple beneficial effects of tissue protection, anti-inflammatory, antioxidant, and antiapoptosis, especially natural active substances, are expected to play a protective role in ameliorating intestinal I/R injury.

Sesamin is found to protect against diverse tissue injuries, including brain injury [10, 57], liver injury [58–62], lung injury [63, 64], and renal injury [7, 65, 66]. Sesamin is also found to have diverse biological effects in amelioration of inflammation [4, 5, 67, 68], oxidation [4, 5, 67], and apoptosis [60, 61, 66, 68]. These studies indicate that sesamin may play a potential role in ameliorating intestinal injury, especially intestinal I/R injury. And our study also confirmed this potential role of sesamin.

Our results showed that sesamin significantly alleviated rat intestinal I/R injuries and reduced the injury-related serum biochemical indicators (ALT, AST) in rats. Sesamin protected against and ameliorated intestinal I/R injuries through its anti-inflammatory, antioxidative, and antiapoptotic effects with the following features. Sesamin-induced anti-inflammatory effects were characterized by reducing proinflammatory cytokines, including TNF-*α*, IL-6, and IL-1*β* in the serum of I/R-injured rats, and the activity of MPO in the I/R-injured intestinal tissues of rats. Sesamin-induced antioxidant effects were mediated via downregulating lipid peroxidation (MDA) and upregulating GSH and SOD in I/R-injured intestinal tissues and decreasing ROS generation and oxidation in H/R-injured IEC-6 cells. Sesamin-reduced apoptosis was characterized by decreasing the TUNEL-positive cells, downregulating the increased Bax and caspase 3 protein expression, and upregulating the decreased protein expression of Bcl2 in I/R-injured intestinal tissues.

During the last decade, various treatment strategies, such as antioxidative stress, anti-inflammation, and antiapoptosis, have shown promising effects in alleviating intestinal I/R injury [69]. Also, multiple signaling pathways involved in preventing and ameliorating intestinal I/R damage have been gradually discovered, helping us to further understand the relevant mechanisms. For instance (Table 2: supplementary material S2), the ameliorative effects of simvastatin on intestinal I/R injuries are mediated through the regulation of the Omi/HtrA2 signaling pathway [70]. The Jagged-2/Notch-1/Hes-1 signaling pathway participates in the regeneration of intestinal epithelium, increasing the proliferation of crypt epithelial cells [71]. Icariin-induced protective effects against intestinal I/R-induced acute lung injury are mediated through the SIRT1/FOXO3 signaling pathway [72]. Leptin improves the intestinal I/R and peritoneal macrophage H/R damage by enhancing ERK1/2 phosphorylation and promoting the signal pathway related to NO production [73]. Epigallocatechin-3-gallate inhibits the inflammatory response by activating the PI3K/Akt signaling pathway, thereby significantly reducing acute intestinal I/R in rats [74]. Paeoniflorin shows a protection on rat intestinal I/R injuries through reducing tissue inflammation and oxidative stress and alleviating the autophagy flux of intestinal I/R impaired by the activation of the LKB1/AMPK signaling pathway [22]. Myricetin ameliorates intestinal I/R injury through inhibiting inflammation and oxidation and reducing the apoptosis by inhibiting the MKK7/JNK signaling pathway [16].

Sesamin also shows its potential role in protecting and ameliorating injuries, and diverse signaling pathways are related (Table 3: supplementary material S3). Sesamin reduces apoptosis and inflammation after experimental myocardial infarction by inhibiting JNK and NF-*κ*B pathways [68]. Sesamin attenuates lipopolysaccharide-induced acute lung injury via inhibition of the TLR4 signaling pathway [64], enhances Nrf2-mediated protective defense against oxidative stress and inflammation in colitis by activating AKT and ERK [75], protects against renal I/R injury by promoting the CD39-adenosine-A2AR signal pathway [7], inhibits CCl_4_-induced oxidative stress-mediated apoptosis in mice via the JNK pathway [60], and ameliorates myocardial I/R injuries through activation of the Akt/eNOS signaling pathway [8].

Among the multiple signaling pathways involved in ameliorating injuries, evidence indicates that activation of the Nrf2 signaling pathway mediates the amelioration of the injured tissues through inhibiting inflammation, oxidative stress, and apoptosis. Polydatin attenuates spinal cord injury in rats by inhibiting oxidative stress and microglia apoptosis via activating the Nrf2/HO-1 pathway, which can be neutralized by silencing Nrf2 using specific siRNA [76]. Nrf2 knockdown in traumatic brain injury mice blunts aucubin-induced the antioxidant and anti-inflammatory neuroprotective effects [77]. GRP78 effectively protects against hypoxia/reperfusion-induced myocardial apoptosis via promotion of the Nrf2/HO-1 signaling pathway, and blockade of Nrf2/HO-1 signaling by the HO-1 inhibitor zinc protoporphyrin IX (Znpp) significantly retrieves H9c2 cell apoptosis [78]. Knockdown of Nrf2 by short-hairpin RNA abrogates resveratrol-mediated protective effects against apoptosis and antioxidant gene expression induced by H_2_O_2_ [79]. Nrf2 inhibitor ML385 weakens the ameliorative effects of rosmarinic acid on oxidant stress, inflammation, and apoptosis in spinal cord injury rats [80].

Our results indicated that the protein expression of Nrf2, HO-1, and NQO1 was significantly increased and further increased in the presence of sesamin in both I/R-injured intestinal tissues and H/R-injured IEC-6 cells. In accordance with the sesamin-increased protein expression of Nrf2/HO-1/NQO1, our study indicated that overexpression of Nrf2 via using Nrf2 cDNA transfection also significantly increased the expression of Nrf2, HO-1, and NQO1 in H/R-injured IEC-6 cells, and sesamin+Nrf2 cDNA further increased Nrf2/HO-1/NQO1 in H/R-injured IEC-6 cells. On the contrary, Nrf2 siRNA and Nrf2 inhibitor (ML385) significantly reduced the protein expression of Nrf2, HO-1, and NQO1, respectively, in H/R-injured IEC-6 cells. Nrf2, which contains the antioxidant genes of the upstream ARE sequence (such as HO-1), is regarded as the main regulator of oxidative stress [81–86]. Our results confirmed the role of Nrf2 in reduction of oxidative stress: sesamin-induced amelioration of oxidative stress was reversed by using Nrf2 inhibitor ML385. And these results are consistent with sesamin-induced protection against I/R injured intestinal tissues and H/R-injured IEC-6 cells.

In conclusion, our study indicated that sesamin pretreatment significantly protected against and ameliorated intestinal histological damage, excessive oxidative stress, inflammation, and apoptosis caused by intestinal I/R. And our study suggests that Nrf2/HO-1/NQO1 signaling pathway activation is involved in sesamin-induced inhibition of inflammation, oxidative stress, and apoptosis in ameliorating intestinal I/R injuries. Our study indicates that sesamin can be considered a prophylactic drug for ameliorating intestinal I/R injuries, and also suggests a potential target for therapeutic intervention to counteract gut ischemic diseases in translational research. As multiple signaling pathways are involved in sesamin-induced injury amelioration, the exact relationship among these signaling pathways in sesamin-induced amelioration of intestinal injuries remains to be clarified.

## Data Availability

All data used to support the findings of this study are included within the article.

## Abbreviations

I/R: Ischemia-reperfusion
H/R: Hypoxia/reoxygenation
SE: Sesamin
SD: Sprague-Dawley
IEC-6: Intestinal epithelial cells-6
SMA: Superior mesenteric artery
AST: Aspartate aminotransferase
ALT: Alanine aminotransferase
H&E: Hematoxylin and eosin
MPO: Myeloperoxidase
IL-6: Interleukin-6
IL-1*β*: Interleukin-1*β*
TNF-*α*: Tumor necrosis factor-*α*
SOD: Superoxide dismutase
GSH: Glutathione
MDA: Malondialdehyde
Bax: Bcl-2-associated X
Bcl-2: B-cell lymphoma-2
Caspase-3: Cysteinyl aspartate specific proteinase 3
Nrf2: Nuclear factor erythroid 2-related factor 2
HO-1: Heme oxygenase 1
NQO1: NAD(P)H quinone dehydrogenase 1
PBS: Phosphate-buffered saline
TUNEL: Terminal deoxynucleotidyl transferase- (TdT-) mediated dUTP nick-end labeling
DAPI: 4′,6-Diamidino-2-phenylindole
DMEM: Dulbecco's minimal essential medium
FBS: Fetal bovine serum
CCK-8: Cell counting kit-8
CMC-Na: Sodium carboxymethyl cellulose
ROS: Reactive oxygen species
SDS-PAGE: Sodium dodecyl sulfate polyacrylamide gel electrophoresis
PVDF: Polyvinylidenefluoride
TBS-T: Tris-buffered saline and Tween-20
ECL: Enhanced chemiluminescent
SEM: Standard error of mean.

## Supplementary Materials

Supplementary Materials S1 Table 1: chemicals and materials used in this study and their manufacturer. S2 Table 2: examples of signaling pathways that can protect against and ameliorate intestinal I/R injury. S3 Table 3: examples of signaling pathways related to sesamin-ameliorated injuries.
