# Supplementary material for: Sesamin Protects against and Ameliorates Rat Intestinal Ischemia/Reperfusion Injury with Involvement of Activating Nrf2/HO-1/NQO1 Signaling Pathway
Source: Oxid Med Cell Longev. 2021 Sep 29;2021:5147069. doi: 10.1155/2021/5147069 (PMC8494576; doi:10.1155/2021/5147069)
Supplement: Supplementary Materials — S1 Table 1: chemicals and materials used in this study and their manufacturer. S2 Table 2: examples of signaling pathways that can protect against and ameliorate intestinal I/R injury. S3 Table 3: examples of signaling pathways related to sesamin-ameliorated injuries. [file 5147069.f1.docx]

## ID 5147069

## Sesamin protects against and ameliorates rat intestinal ischemia/reperfusion injury with involvement of activating Nrf2/HO-1/NQO1 signaling pathway

**Supplementary material**

**S1:**

**Table 1. Chemicals and materials used in this study and their manufacturer.**

| Chemicals and materials | Manufacturer |
| --- | --- |
| sesamin | Dalian Meilun Biotech Co., Ltd. |
| AST, ALT, MPO, MDA, SOD, GSH test kits, and ML385 | Nanjing Jiancheng Institute of Biotechnology |
| IL-1β, IL-6, TNF-α test kits | ShangHai Lengton Bioscience Co. Ltd |
| DMEM | Hylone Laboratory |
| FBS | Gibco |
| TUNEL kit | Servicebio Company |
| CCK-8 kit | Bio-Tool |
| siRNA of Nrf2 and Lipo-fectamine 2000 | Gene Pharma |
| IEC-6 cells | American Tissue Culture Collection |
| others | Sigma-Aldrich |

**S2:**

**Table 2. Examples of signaling pathways that can protect against and ameliorate intestinal I/R injury.**

| Compound | Signaling pathway  (Up-regulate ↑ or down-regulate ↓) | Effects | Animals/  cell lines | Reference |
| --- | --- | --- | --- | --- |
| simvastatin | Omi/HtrA2 ↓ | decreased oxidative stress, inflammatory damage, and apoptosis | SD rats | 70 |
| - | Jagged-2/Notch-1/  Hes-1 ↑ | increased the proliferation of crypt epithelial cells and participated in the early intestinal epithelial regeneration after I / R injury | SD rats; IEC-6 cells | 71 |
| icariin | SIRT1 ↑ | modulation of downstream antioxidative and anti-apoptotic factors | SD rats | 72 |
| leptin | ERK1/2 ↑ | increased NO production in the intestines, and increased the NO and total antioxidative capacity levels in cells | mice; murine peritoneal macrophage | 73 |
| epigallocatechin-3-gallate | PI3K/Akt ↑ | suppressed inflammatory response | Wistar rats | 74 |
| paeoniflorin | LKB1/AMPK ↑ | reduced tissue inflammation, oxidative stress and alleviated the autophagy flux | SD rats; IEC-6 cells | 22 |
| myricetin | MKK7/JNK ↓ | inhibited inflammation, oxidation and reduced the apoptosis | SD rats; IEC-6 cells | 16 |

**S3:**

**Table 3. Examples of signaling pathways related to sesamin-ameliorated injuries.**

| Injury type | Signaling pathway  (Up-regulate ↑ or down-regulate ↓) | Effects | Animals/  cell lines | Reference |
| --- | --- | --- | --- | --- |
| myocardial infarction -induced cardiac damage | JNK and NF-κB ↓ | decreased myocardial apoptosis and inflammatory response | mice | 68 |
| LPS-induced acute lung injury | TLR4 ↓ | suppressed LPS-induced inflammatory cytokines | mice | 64 |
| dextran sulfate sodium (DSS)- induced ulcerative colitis | AKT/ERK ↑; Nrf2 ↑ | defense against oxidative stress and inflammation | mice; Caco-2 cells | 75 |
| renal ischemia reperfusion injury | CD39-adenosine-A2AR ↑ | inhibited inﬂammatory responses | mice | 7 |
| CCl4-induced liver injury | JNK ↑ | protected against CCl_4_-induced oxidative stress-mediated apoptosis | mice | 60 |
| myocardial ischemia reperfusion injury | Akt/eNOS ↑ | enhanced antioxidant capacity, increased NO synthesis, and suppressed cardiac myocyte apoptosis | SD rats | 8 |
